# Prevalence and antimicrobial susceptibility patterns of extended spectrum beta-lactamase producing *Entrobacteriaceae* in the University of Gondar Referral Hospital environments, northwest Ethiopia

**DOI:** 10.1186/s13104-018-3443-1

**Published:** 2018-05-22

**Authors:** Tigist Engda, Feleke Moges, Aschalew Gelaw, Setegn Eshete, Feleke Mekonnen

**Affiliations:** Department of Microbiology, School of Biomedical and Laboratory Sciences, College of Medicine and Health Sciences, University of Gondar, P. O. Box 196, Gondar, Ethiopia

**Keywords:** ESBL, *Entrobacteriaceae*, Hospital environments

## Abstract

**Objective:**

This study aimed at assessing the magnitude, distribution, and the antimicrobial susceptibility of the extended spectrum beta-lactamase producing *Entrobacteriaceae* in the University of Gondar Referral Hospital environments.

**Results:**

Out of a total of 384 samples, 14.8% were ESBL producing *Entrobacteriaceae*, where 42.10% *Klebsiella pneumoniae*, 35.09% *Escherchia coli* and 7.01% *Proteus mirabilis* were the predominant isolates. Most ESBL producing isolates, that is, 24.56, 22.8, and 22.8% were found from waste water, sinks and bedside tables respectively. All ESBL producing *Entrobacteriaceae* were found to be resistant to ceftriaxone, ceftazidime, cefpirome, cefpodoxime, and amoxicillin with Clavulanic acid. Resistance rate was also high for non-beta-lactam antimicrobials, like chloramphenicol (70.18%), cotrimoxazole (64.91%), norfloxacin (42.10%), ciprofloxacin (43.86%), and gentamicin (19.30%).

## Introduction

Hospital environment is a potential source of nosocomial infections, since it houses both patients with diverse pathogenic microorganism and a large number of susceptible individuals [1–3].

The ever-increasing bacterial resistance to antibiotics is one of the most thought-provoking tasks of medical issues today [4]. A persistent exposure of bacterial strains to a multitude of beta-lactam antibiotics has induced a dynamic, continuous production and mutation of beta-lactamase in the bacteria leading to the development of extended spectrum beta-lactamases (ESBLs) causing resistance to broad spectrum beta-lactam antibiotics [5–7]. ESBL is a plasmid-mediated enzyme able to hydrolyze all beta-lactams except cephamycin and carbapenems, and constitute a large heterogeneous group of enzymes with different origins [5, 8].

An extended spectrum beta-lactamase production has been observed mostly in *Entrobacteriaceae,* and their resistance increased mainly due to the spreading of ESBL and the emergence of different genes [9–11]. The frequently ESBL producing *Entrobacteriaceae* are *Escherichia coli, Klebsiella pneumoniae* and *Proteus species*. However, all other clinically relevant *Entrobacteriaceae* species are also common ESBL producers. The distribution of ESBL depends on geographic locality, hospital wards, groups of patients and the type of infection [12–16].

The resistance patterns of ESBLs were initially considered as a problem related to nosocomial outbreak, mainly in Intensive Care Units, surgical procedures, bladder catheterization, long-term hospitalization, and frequent exposure of broad spectrum antimicrobials. However, the distribution of ESBL producing *Entrobacteriaceae* becomes common in any other area of the hospital indicating a high possibility of horizontal gene transfer among strains of different genera within the hospital environment [17–21].

Reports about ESBLs producing *Entrobacteriaceae* at hospital environments in northwest Ethiopia are limited. Thus, this study was carried out to assess and determine the rate of ESBLs producing *Entrobacteriaceae* and their antimicrobial susceptibility patterns in the hospital environment at the University of Gondar Referral Hospital.

## Main text

### Methods

#### Study design and area

A hospital-based cross-sectional study was conducted at the University of Gondar Referral Hospital from January to June 2014. The hospital provides surgical, medical, gynecologic and obstetric, and ophthalmologic services to over 5 million inhabitants. It has different wards with 465 beds and outpatient departments.

#### Sample size

Seven sample selection sites were identified prior to data collection. The simple random sampling technique was applied. Out of the 2508 total population, 384 were included in the study based on the single population proportion formula.

#### Sampling techniques

Samples were collected from bed frames, bedside tables, door handlers, floors, sinks, waiting chairs, walls, and the waste water of the hospital which have direct contact with patients, their families, and health care providers. Medical wards, surgical wards, the Gynecology and Obstetrics ward, the Fistula clinic, the Eye clinic, the hospital café, and hospital sewages were the study sites. Sterile cotton tipped swabs moistened with normal saline were rotated against the surfaces of inanimate objects to obtain specimens, and waste water was collected from the hospital sewage by a sterile screw capped bottle and transported to the microbiology laboratory. Each sample was transferred to the selective media, Hicrome ESBL agar base, to assess the ESBL production in 2 h. The species was differentiated by specific colony color and again by a biochemical test [22, 23]. Antimicrobial susceptibility tests were conducted using the disc diffusion method for beta-lactam and non-beta-lactam antimicrobials [24–26].

### Data analysis

The descriptive statistical analysis was performed by using SPSS version 20 software program.

## Results

### Distribution of ESBL producing *Entrobacteriaceae* in the hospital environment (Table 1)

ESBL producing *Entrobacteriaceae,* namely *Escherchia coli*, *Klebsiella pneumoniae*, *Klebsiella ozenae, Proteus mirabilis, Entrobacter cloceae, Entrobacter aerogens, Citrobacter* and *Providencia stuarti* were isolated from the samples. The predominant species were *Klebsiella pneumoniae* (42.10%), *Escherchia coli* (35.09%) and *Proteus mirabilis* (7.01%). *Escherchia coli* and *Klebsiella pneumoniae* were found in all the selected types of samples. However, *Klebsiella ozenae* were found in sinks; *Proteus mirabilis* in bed frames, floor and sinks; *Entrobacter cloace* in bed frame; *Entrobacter aerogens* in bed tables and bed frames; *Citrobacter* in floor and sinks and *Providencia stuarti* in bed frames and sinks (Table 2).

**Table 1:** Profile of ESBL producing *Entrobacteriaceae* in different hospital environmental samples at the University of Gondar Referral Hospital, January–June 2014

ESBL producing *Entrobacteriaceae*	Types of samples								
	Bed side table N = 70 (N, %)	Bed frame N = 69 (N, %)	Floor N = 69 (N, %)	Wall N = 69 (N, %)	Door handler N = 34 (N, %)	Waiting chair N = 31 (N, %)	Sink N = 22 (N, %)	Waste water N = 15 (N, %)	Café chair N = 5 (N, %)
*E. coli*	3 (4.3)	3 (4.3)	1 (1.4)	1 (1.4)	1 (2.9)	1 (3.2)	3 (13.6)	7 (46.7)	0 (0.0)
*K. pneumoniae*	8 (11.4)	2 (2.9)	1 (1.4)	1 (1.4)	0 (0.0)	1 (3.2)	4 (18.1)	7 (46.7)	0 (0.0)
*K. ozenae*	0 (0.0)	0 (0.0)	0 (0.0)	0 (0.0)	0 (0.0)	0 (0.0)	2 (3.5)	0 (0.0)	0 (0.0)
*P. mirabilis*	1 (1.4)	1 (1.4)	0 (0.0)	0 (0.0)	0 (0.0)	0 (0.0)	2 (9.0)	0 (0.0)	0 (0.0)
*E. cloace*	0 (0.0)	1 (1.4)	0 (0.0)	0 (0.0)	0 (0.0)	0 (0.0)	0 (0.0)	0 (0.0)	0 (0.0)
*E. aerogens*	1 (1.4)	1 (1.4)	0 (0.0)	0 (0.0)	0 (0.0)	0 (0.0)	0 (0.0)	0 (0.0)	0 (0.0)
*Citrobacter*	0 (0.0)	0 (0.0)	1 (1.4)	0 (0.0)	0 (0.0)	0 (0.0)	1 (1.8)	0 (0.0)	0 (0.0)
*P. stuart*	0 (0.0)	1 (1.4)	0 (0.0)	0 (0.0)	0 (0.0)	0 (0.0)	1 (4.5)	0 (.0)	0 (0.0)
Sum = 57	13 (22.8)	9 (13.0)	3 (4.3)	2 (2.9)	1 (2.9)	2 (3.5)	13 (59.0)	14 (24.6)	0 (0.0)

ESBL producing *Entrobacteriaceae* were isolated mainly from the medical ward (52.63%), sewage (24.56%) and surgical ward (10.53%). No *Entrobacteriaceae* were isolated from hospital café chairs (Table 2)

**Table 2:** The distribution of ESBL producing *Entrobacteriaceae* in different sites of the hospital environment at the University of Gondar Referral Hospital, northwest Ethiopia, January–June 2014

Bacterial isolates	Sites of environmental samples						
	Medical ward n = 188 (N, %)	Surgical ward n = 70 (N, %)	Gyn-obs ward n = 52 (N, %)	Fistula clinic n = 29 (N, %)	Eye clinic n = 25 (N, %)	Sewage n = 15 (N, %)	Café chairs n = 5 (N, %)
*E. coli*	12 (6.3)	1 (1.4)	0 (0.0)	0 (0.0)	0 (0.0)	7 (46.7)	0 (0.0)
*K. pneumoniae*	14 (7.4)	1 (1.4)	1 (1.9)	0 (0.0)	1 (4.0)	7 (46.7)	0 (0.0)
*K. ozenae*	1 (0.5)	1 (1.4)	0 (0.0)	0 (0.0)	0 (0.0)	0 (0.0)	0 (0.0)
*P. mirabilis*	2 (1.0)	1 (1.4)	0 (0.0)	1 (3.4)	1 (4.0)	0 (0.0)	0 (0.0)
*E. cloacae*	0 (0.0)	0 (0.0)	1 (1.9)	0 (0.0)	0 (0.0)	0 (0.0)	0 (0.0)
*E. aerogens*	0 (0.0)	0 (0.0)	0 (0.0)	0 (0.0)	2 (8.0)	0 (0.0)	0 (0.0)
*Citrobacter spp*	1 (0.5)	1 (1.4)	0 (0.0)	0 (0.0)	0 (0.0)	0 (0.0)	0 (0.0)
*P. stuart*	0 (0.0)	1 (1.4)	1 (1.9)	0 (0.0)	0 (0.0)	0 (0.0)	0 (0.0)
Total	30 (52.6)	6 (10.5)	3 (5.3)	1 (1.8)	3 (5.3)	14 (24.6)	0 (0.0)

The highest number of ESBL producing *Entrobacteriaceae* isolates were found from sinks (22.80%), bed tables (22.80%), waste water (15.79%) and less than 5% were found in bed frames, door handles, floors, walls, and waiting chairs. However, no isolates were found from hospital cafe chairs.

### Antimicrobial susceptibility patterns of ESBL producing Entrobacteriaceae isolates from the hospital environment (Table 3)

All ESBL producing *Entrobacteriaceae* were found to be 100% resistant to cefpirome, cefpodoxime, ceftazidime, ceftriaxone, and amoxicillin with clavulanic acid. However, 70.18, 64.91, 42.10, 43.86, and 19.30% were resistant to chloramphenicol, cotrimoxazole, norfloxacin, ciprofloxacin, and gentamicin, respectively.

**Table 3:** Antimicrobial susceptibility pattern of ESBL producing *Entrobacteriaceae* isolated from the hospital environment at the University of Gondar Referral Hospital, northwest Ethiopia, and January–June 2014

Organism	Pattern	Antimicrobial agent’s N (%)									
		CFP (N, %)	CPD (N, %)	CAZ (N, %)	CTR (N, %)	CIP (N, %)	NX (N, %)	GEN (N, %)	C (N, %)	AMC (N, %)	SXT (N, %)
*E. coli*	S	0 (0.0)	0 (0.0)	0 (0.0)	0 (0.0)	14 (70.0)	15 (75.0)	16 (80.0)	9 (45.0)	0 (0.0)	13 (65.0)
	R	20 (100)	20 (100)	20 (100)	20 (100)	6 (30.0)	5 (25.0)	4 (20.0)	11 (55.0)	20 (100)	7 (35.0)
*K. pneumonia*	S	0 (0.0)	0 (0.0)	0 (0.0)	0 (0.0)	13 (54.2)	13 (54.2)	18 (75.0)	8 (33.3)	0 (0.0)	2 (8.3)
	R	24 (100)	24 (100)	24 (100)	24 (100)	11 (45.8)	11 (45.8)	6 (25.0)	16 (66.7)	24 (100)	22 (91.7)
*K. ozenae*	S	0 (0.0)	0 (0.0)	0 (0.0)	0 (0.0)	2 (100.0)	2 (100.0)	2 (100.0)	0 (0.0)	0 (0.0)	0 (0.0)
	R	2 (100)	2 (100)	2 (100)	2 (100)	0 (0.0)	0 (0.0)	0 (0.0)	2 (100)	2 (100)	2 (100)
*P. mirabilis*	S	0 (0.0)	0 (0.0)	0 (0.0)	0 (0.0)	3 (75.0)	3 (75.0)	4 (100)	0 (0.0)	0 (0.0)	2 (50.0)
	R	4 (100)	4 (100)	4 (100)	4 (100)	1 (25.0)	1 (25.0)	0 (0.0)	4 (100)	4 (100)	2 (50.0)
*E. cloace*	S	0 (0.0)	0 (0.0)	0 (0.0)	0 (0.0)	0 (0.0)	0 (0.0)	1 (100)	0 (0.0)	0 (0.0)	0 (0.0)
	R	1 (100)	1 (100)	1 (100)	1 (100)	1 (100)	1 (100)	0 (0.0)	1 (100)	1 (100)	1 (100)
*E. aerogens*	S	0 (0.0)	0 (0.0)	0 (0.0)	0 (0.0)	0 (0.0)	0 (0.0)	2 (100)	0 (0.0)	0 (0.0)	0 (0.0)
	R	2 (100)	2 (100)	2 (100)	2 (100)	2 (100)	2 (100)	0 (0.0)	2 (100)	2 (100)	2 (100)
*Citrobacter*	S	0 (0.0)	0 (0.0)	0 (0.0)	0 (0.0)	0 (0.0)	0 (0.0)	2 (100)	0 (0.0)	0 (0.0)	0 (0.0)
	R	2 (100)	2 (100)	2 (100)	2 (100)	2 (100)	2 (100)	0 (0.0)	2 (100)	2 (100)	2 (100)
*P. stuart*	S	0 (0.0)	0 (0.0)	0 (0.0)	0 (0.0)	0 (0.0)	0 (0.0)	1 (50.0)	0 (0.0)	0 (0.0)	0 (0.0)
	R	2 (100)	2 (100)	2 (100)	2 (100)	2 (100)	2 (100)	1 (50.0)	2 (100)	2 (100)	2 (100)
Total (57)	S	0 (0.0)	0 (0.0)	0 (0.0)	0 (0.0)	32 (56.1)	33 (57.9)	46 (80.7)	18 (31.6)	0 (0.0)	17 (29.8)
	R	57 (100)	57 (100)	57 (100)	57 (100)	25 (43.9)	24 (42.1)	11 (19.3)	39 (68.4)	57 (100)	40 (70.2)

*CFP* cefpirome, *CPD* cefpodoxime, *CAZ* ceftazidime, *CTR* ceftriaxone, *CIP* ciprofloxacin, *NX* norfloxacin, *GEN* gentamycin, *C* chloramphenicol, *SXT* sulfamethoxazole + trimethoprim **(**cotrimoxazole), *AMC* amoxicillin with clavulanic acid, *S* sensitive, *R* resistance

*Klebsiella pneumoniae* were resistant to 91.7% cotrimoxazole, 66.7% chloramphenicol, 45.8% norfloxacin, 45.8% ciprofloxacin, and 25.0% gentamicin. *Escherchia coli* was also resistant to chloramphenicol (55.0%), cotrimoxazole (35.0%), norfloxacin (25.0%), ciprofloxacin (30.0%), and gentamicin (20.0%).

Of the total ESBL producing *Proteus mirabilis*, only 25.0% strain was resistant to norfloxacin, ciprofloxacin, and cotrimoxazole. But, all isolates were susceptible to gentamicin.All isolates of *Klebsiella ozenae* were resistant to chloramphenicol, and cotrimoxazole, whereas all isolates were sensitive to norfloxacin, ciprofloxacin, and gentamicin.

### Multiple antibiotics resistance

More than 56% of the ESBL producing *Entrobacteriaceae* isolates demonstrated multiple non-beta lactam antibiotics resistance against ciprofloxacin, norfloxacin, gentamicin, chloramphenicol, and cotrimoxazole, of which *Klebsiella pneumoniae* (58%) and *Escherchia coli* (40%) showed a high level of multiple drug resistance. Despite the fact that the number of isolates was small; *Entrobacter cloceae*, *Entrobacter aerogens*, *Citrobacter,* and *Providencia stuart* were multidrug resistant.

## Discussion

The rise of bacterial pathogens in hospital environment is associated with an increasing in nosocomial infections. *Entrobacteriaceae* is one of the most important causes of nosocomial and community acquired infections where Beta-lactam antibiotics are the first choice for treatment of infections caused by *Entrobacteriaceae.* However, they produce extended spectrum beta-lactamases (ESBLs) that cause high resistance to the beta-lactam antibiotics. Of the different genera of *Entrobacteriaceae*, Escherichia coli is the leading urinary tract pathogens with septicemic potential, *Klebsiella pneumonia* causes lobar pneumonia and often outbreaks in hospital settings, *Proteus mirabilis* cause urinary tract infections, chronic ear infection and septicaemia, *Klebsiella ozane* cause atrophic rhinitis*, Enterobacter* causes urinary tract infection & sepsis and *Citrobacter* Causes urinary tract infection, sepsis, wound infection, osteomyelitis in elderly hospitalized patients and neonatal meningitis [1].

Routine ESBLs detection is not common in many developing countries including Ethiopia. Hence, assessing the prevalence and the drug susceptibility of ESBL producing bacteria is very important to develop guideline for the management of infections associated with such organisms. This study was carried out to examine the prevalence and rate of antimicrobial drug resistance of ESBL producing *Entrobacteriaceae* at the University of Gondar Referral Hospital environments. The data presented in this study will provide information of immediate public health importance to clinicians on the selection of antimicrobial agents for patients suffering from infections caused by ESBL *Entrobacteriaceae* in northwest Ethiopia.

According to this study, the total ESBL producing *Entrobacteriaceae* were 14.8% of which the predominant isolate was *Klebsiella pneumoniae* (14.7%) which is in line with a study finding in Alexandria, Egypt (14.9%) [27] but lower than those of studies conducted in France (37%), Algeria (44.5%) [28, 29] and Upper Egypt (56.25%) [30]. these variations might be due to the number of patients that attended each hospital, disease exposure, and geographic differences among the study areas. The second predominant ESBL producing organism was *Escherchia coli* (12.3%) which is higher than those of studies conducted in France (5%) and Algeria (4%) [28, 29]. However, it is lower than the report of studies in Alexandria, Egypt (85%) and Upper Egypt (43.75%) [27, 30]. These differences may be due to differences in the type of health care activities and infection control practices in the hospitals. The result of *Entrobacter cloceae* (1.2%) was lower than those of a research carried out in Algeria’s Intensive Care Unit of the hospital (11%) [29].

In our findings, inanimate objects in the hospital OPD, wards, surgical room, delivery room, waiting area, and the waste water from different sewage systems were variously contaminated by multiple drug resistant ESBL producing bacteria. Moreover, medical wards, sewage, and the surgical ward had 52.6, 24.6, and 10.5% of ESBL producing *Entrobacteriaceae* respectively, the overall distribution of ESBL producing *Entrobacteriaceae* in different sections was higher than the report in France [28]. This may be due to differences in the number of patients attending each section of the hospitals, and because the value of ESBL producing organisms in different sites is directly related to the type of samples taken.

Different studies also showed that the proportion of organisms which cause environmental contamination is directly associated to the number of patients who visit the hospitals. According to this and a previous study more hospital environment contamination was caused by ESBL producing *Klebsiella pneumoniae* than ESBL producing *Escherchia coli* in an *Entrobacteriaceae* family [28, 31, 32].

In an antimicrobial susceptibility test, all isolates of ESBL producing *Entrobacteriaceae* were 100% resistant to *cefpirome*, *cefpodoxime, ceftazidime, ceftriaxone* and *amoxicillin* with *clavulanic acid w*hich is much higher than reports from Poland, cefpodoxime(73.5%) and ceftazidime(81.6%) [33], and Upper Egypt, *Klebsiella pneumonia* (95.5%) for *ceftazidime* and *Escherchia coli* (91.4%) for *ceftazidime* [32].

Even though the rate of resistance was low for non-beta lactam antibiotics compared to beta-lactam antibiotics, ESBL producing *Entrobacteriaceae* demonstrated an alarming rate of resistance. In this finding, the rate of resistance to *Klebsiella pneumoniae* was 25.0% gentamicin and 45.8% ciprofloxacin which is lower than that of a study done in Upper Egypt where gentamicin was 84.4% and ciprofloxacin 77.7% [32]. Moreover, *Escherchia coli* isolates showed 20% resistance to gentamicin and 30% to ciprofloxacin. This result shows a lower resistance rate compared to the study done in Upper Egypt (42.8%) to gentamicin and (68.5%) to ciprofloxacin [32].

The most challenging condition in the management of infectious diseases associated with ESBL producing *Entrobacteriaceae* are the development of multiple drug resistance (Resistant to two or more drugs). There is a report on co-resistance of ESBL producing *Entrobacteriaceae*, but not to more than three antibiotics [30]. However, the current study showed that a high frequency of multiple antibiotics resistance to commonly used antibiotics. This might be due to inappropriate use of antimicrobials, lack of laboratory diagnostic tests, failure of patient adherence to their medication, and unavailability of guidelines for the selection of antibiotics.

Based on our findings, the hospital should install a proper hygiene and rational use of antimicrobial activities in order to control and prevent the possibility of spreading of infectious diseases in the compound. Moreover; the sewage system of the hospital should be managed to decrease environmental contaminations.

## Limitation of the study

Some type of hospital environments were not included in the study. So to get good distribution of ESBLs *Entrobacteriaceae* in hospital environment, it would be better assessing all types of the hospital environments. It would have a better figurative if the study was conducted in different hospitals that are found in north-west Ethiopia.

## Abbreviations

CTX-M: Cefotaximase Munich
CLSI: Clinical Laboratory Standard Institute
DDST: double-disc synergy test
ESBL: extended spectrum beta lactamase
HAIs: health care associated infections
MIC: minimum inhibitory concentration
OXA: oxacillin
SHV: sulphydryl variable
SPSS: statistical package for social sciences
TEM: temoneira
OPD: outpatient department
